# Editorial: Vascular function and mechanisms of aging: hypertension, obesity and metabolic disorders

**DOI:** 10.3389/fphys.2023.1339619

**Published:** 2024-01-04

**Authors:** Eduardo Nava, Andreea Trache, Alejandro Diaz, Antonio Colantuoni

**Affiliations:** ^1^ Department of Medical Sciences, University of Castille-La Mancha School of Medicine, Albacete, Spain; ^2^ Department of Medical Physiology, Texas A&M University Health Science Center, Bryan, United States; ^3^ Department of Biomedical Engineering, Texas A&M University, College Station, United States; ^4^ National University of Central Buenos Aires, Buenos Aires, Argentina; ^5^ University of Naples Federico II Medical School, Naples, Italy

**Keywords:** vascular function, aging, hypertension, obesity, metabolic syndrome

Within the context of age-related decline, quality of life is intensely affected by the functional deterioration of the cardiovascular system. Understanding the role of obesity, hypertension, and metabolic disorders on vascular function and subsequent vascular aging is crucial for clinical and basic research and raises a strong interest to the scientific community. Indeed, a quick search yielded 26,984 articles published in the PUBMED database with the terms “Vascular Function and Aging.” Vascular aging is a multifactorial phenomenon that begins even before birth. However, we still do not have a single “true and complete” measure of this complex phenomenon; many parameters and tools have been proposed, all of which describe in a very fragmented way different aspects of the underlying process of “vascular aging” (Rietzschel and De Buyzere, 2022). Aging, hypertension, insulin resistance, obesity and atherosclerosis severely deteriorate the morphology and function of the cardiovascular system (Folkow and Svanborg, 1993; Küng and Lüscher, 1995). Blood vessels, specifically, show mechanical and morphological changes as well as endothelial and smooth muscle dysfunction (Ferrari et al., 2003). A decline in the distensibility of the aorta (Hallock and Benson, 1937) can be often detected in aging (Reddy et al., 2003). The morphological bases for these mechanical alterations are the vascular changes that take place during senescence. Arteries become elongated and tortuous presenting intimal and medial thickening as well as an increase in collagen accumulation in the media, and elastin fragmentation (Ferrari et al., 2003). Arterial stiffness is a marker of the interactions between hemodynamics and mechanosensing. Despite advances in studying age-induced arterial stiffness (Sehgel et al., 2015; Trache et al., 2020), there is a need to expand our understanding of the many steps involved in integrin-based adhesion and nucleo-cytoskeletal coupling, as well as mechanotransduction-regulated epigenetics (Lacolley et al., 2020).

Importantly, aging causes a decay in endothelium-dependent relaxation both in animal models (Llorens et al., 2007) and humans (Egashira et al., 1993). Indeed, it can be stated that the cardiovascular disease conditions that convey within the metabolic syndrome, i.e., hypertension, insulin resistance, obesity or atherosclerosis, accelerate the age-induced vascular alterations (Susic et al., 1998). This Research Topic addresses these diseases in the context of aging.

Raberin et al. investigated endothelial function, blood viscosity and cardiovascular disease (CVD) prevalence in elderly males and females. CVD is one of the leading causes of mortality in this population, with middle-aged males being at higher risk than females. After menopause, however, females are no longer protected by hormones. Analyses performed on 182 elderly participants investigated parameters of the endothelial function, estimated by hyperemic response after 5-min arterial occlusion, blood viscosity, HDL (high-density lipoprotein), hematocrit and red blood cell (RBC) aggregation. CVD prevalence was higher in males than in females. The results are really interesting and indicate that females have higher vasoreactivity, characterized by higher hyperemic response to arterial occlusion and higher HDL levels than males. Moreover, blood viscosity was higher in males than in females at any shear rate as well as hematocrit was greater in males than in females, while RBC aggregation did not differ between the two populations. The authors highlight that females present a lower rate of CVD compared to age-matched males, suggesting that females benefit by an increased vascular function and lower blood viscosity.

Dixon and Osei-Owusu investigated the age-related decline in functional elastin, associated with increased arterial stiffness, a known risk factor for developing CVD. While the contribution of elastin insufficiency to the stiffening of conduit arteries is well described, the authors highlight the impact on the structure and function of the resistance vasculature, known to contribute to the total peripheral resistance and the regulation of organ perfusion. Therefore, they assessed how elastin insufficiency affects age-related changes of the structure and biomechanical properties of the renal microvasculature. The authors proposed to clarify how these changes induce variations in renal hemodynamics. Moreover, they were interested in the response of the renal vascular bed to changes in renal perfusion pressure (RPP) in mice: they utilized 4–6-month and 14–16-month-old Eln+/+ and Eln+/− female mice. Using Doppler ultrasonography, the authors found that resistive index and pulsatility index were elevated in young Eln+/− and aged mice. Interestingly, histological examination indicated thinner internal and external elastic laminae, accompanied by increased elastin fragmentation in the medial layer without calcium deposition in the small intrarenal arteries from young Eln+/− and aged mice. Furthermore, pressure myography of interlobar arteries revealed that vessels from the above mentioned population of mice had a slight decrease in distensibility during pressure loading, accompanied by a substantial decline in vascular recoil efficiency upon pressure unloading. The authors investigated renal hemodynamics by clamping neurohumoral input and increased RPP by simultaneously occluding the superior mesenteric and celiac arteries. They observed that increased RPP caused marked changes in blood pressure in all female mice. However, the changes in renal vascular resistance and renal blood flow (RBF) were blunted in young Eln+/− and aged mice, accompanied by decreased autoregulatory index, indicating greater impairment of renal autoregulation. Finally, they report that increased pulse pressure in aged Eln+/− mice positively correlates with high RBF. In conclusion, this work highlights that the loss of elastin negatively affects the structure and function of the renal microvasculature, worsening age-related decline in kidney function.

Aguilar et al. provided a detailed review of the current state of knowledge about the mechanisms that regulate endothelial stiffness in CVD and aging. Arterial stiffness results in molecular and mechanical changes leading to an impaired ability of blood vessels to control blood flow and blood pressure. Endothelial stiffening is critically distinct from vascular wall stiffening that has as major contributors the extracellular matrix and vascular smooth muscle cells. Thus, endothelium stiffening has two distinct physiological roles in the general vessel wall stiffening, disruption of the endothelial barrier and reduction of the endothelial vasodilatory function. First, the authors analyze the contributions of the endothelial cytoskeleton and conclude that endothelium stiffening depends primarily on actomyosin-mediated contractility with possible emerging roles for intermediate filaments. Then, the authors discuss the effects of small RhoA and Rac GTPases activation in response to blood flow, blood pressure, and circumferential stretch. Thus, the formation of stress fibers aligned in the direction of laminar flow is a two-step process in which Rac activation at the cell edge is followed by RhoA activation that stabilizes the elongated cell shape and alignment of actin stress fibers. However, disturbed flow regions result in stress fibers without a determined orientation. In addition, the continuous crosstalk between cells and the extracellular matrix represents an active signalling pathway that also modulates endothelial stiffening. Endothelium stiffness increases when cells are exposed to increased stiffness of the sub-endothelial matrix in aging. However, recent studies from the authors, showed that endothelial stiffening may develop independently of matrix stiffness due to uptake and accumulation of oxidized lipids induced by high fat diet or aging. Lastly, the authors discuss direct evidence of endothelium stiffening in the maintenance of the barrier integrity in disease states, and the role of RhoA-induced contractility in degeneration of VE-cadherin based adherens junctions.

Finally, Zhang et al. examined the association between specific ceramides and metabolic syndrome (MS) risk factors in patients with acute coronary syndrome (ACS) and investigated if ceramides can be used as a substitute for metabolic profiling in this population. Authors have also explored the feasibility of developing a comprehensive medical system that utilizes individual plasma ceramides levels to differentiate MS patients based on their level of severity, particularly in ACS cases. The general conclusion demonstrates a significant link between circulating plasma ceramides and conventional cardiovascular risk factors in ACS patients. Results indicated that Cer18:0 may serve as a valuable surrogate biomarker for metabolic profiles, which can assist in developing preventative treatment strategies for ACS patients. Thus, ceramides may be used to identify patients in early stages of MS and facilitate risk stratification.

In conclusion, the study of vascular function and its relationship with the mechanisms of aging is becoming increasingly important in clinical and basic research with solid evidence and an evolving future. Paraphrasing French writer Charles Augustin Sainte Beuve “Vieillir est encore le seul moyen qu’on ait trouvé de vivre longtemps” (Growing old is still the only way we have found to live for a long time).
